# Prevalence and Clinical Correlates of Sarcopenia and Malnutrition in Lung Transplant Recipients Using EWGSOP2 and GLIM Criteria

**DOI:** 10.3390/nu18152410

**Published:** 2026-07-23

**Authors:** Ioana Muller, Julien Blaess, Margherita Giannini, Camille Daeffler, Veronique Vincent, Anne-Laure Charles, Marianne Riou, Irina Enache, Emmanuel Andrès, Anne Charloux, Alain Meyer, Benjamin Renaud-Picard, Bernard Geny

**Affiliations:** 1 Department of Physiology and Functional Explorations, University Hospital of Strasbourg, 67000 Strasbourg, France; ioana.muller@chru-strasbourg.fr (I.M.); julien.blaess@chru-strasbourg.fr (J.B.); margherita.giannini@chru-strasbourg.fr (M.G.); veronique.vincent@chru-strasbourg.fr (V.V.);; 2 Biomedicine Research Center of Strasbourg (CRBS), UR 3072, “Mitochondria, Oxidative Stress and Muscle Plasticity”, Faculty of Medicine, University of Strasbourg, 67000 Strasbourg, France; 3 Department of Pulmonology, Strasbourg Lung Transplant Program, University Hospital of Strasbourg, 67000 Strasbourg, France; camille.daeffler@chru-strasbourg.fr (C.D.);; 4 Department of Internal Medicine, University Hospital of Strasbourg, 67000 Strasbourg, France; 5 Inserm UMR 1260 “Nanomédecine Regenerative”, University of Strasbourg, 67000 Strasbourg, France

**Keywords:** lung, transplantation, sarcopenia, nutrition, body composition, dual-energy X-ray absorptiometry (DXA), muscle, COPD, ILD

## Abstract

**Background**: Sarcopenia and malnutrition may contribute to persistent functional vulnerability after lung transplantation, yet their prevalence using actual criteria, relationship and clinical correlates remain incompletely defined. **Methods**: Ninety-nine adult lung transplant recipients (Ltx) were included in the study during their routine follow-up. Sarcopenia was defined using EWGSOP2 criteria, integrating muscle strength, dual-energy X-ray absorptiometry-derived appendicular lean mass, and physical performance. Malnutrition was assessed using GLIM criteria before transplantation and at follow-up. Clinical, nutritional, biological, body-composition, and pulmonary-function variables were compared according to sarcopenia status. **Results**: Sarcopenia was identified in 17 recipients (17.2%), including 12 (12.1%) with severe sarcopenia. Malnutrition was frequent before transplantation and persisted at follow-up, affecting 52 of 98 recipients (53.1%) before transplantation and 23 of 99 recipients (23.2%) after transplantation. Sarcopenia was related to underlying diseases leading to transplantation (mainly COPD and ILD) and sarcopenic Ltx were older (66.1 ± 6.7 vs. 57.7 ± 12.6 years; *p* = 0.05), had greater smoking exposure (38.2 ± 25.6 vs. 22.2 ± 20.2 pack-years; *p* = 0.02) and prednisone dose (13.5 ± 8.1 vs. 8.9 ± 2.7 mg/day; *p* = 0.001). Despite a trend towards lower albuminemia (40.5 ± 4.7 vs. 42.1 ± 4.3 g/L; *p* = 0.07), malnutrition was not associated with sarcopenia status. Lung function improved substantially after transplantation, with mean FEV_1_ increasing from 25.7 ± 16.6% to 82.0 ± 25.4% predicted at assessment (*p* < 0.001); however, FEV_1_ at assessment did not differ considering sarcopenia status. **Conclusions**: In lung transplant recipients, EWGSOP2-defined sarcopenia was common and largely uncaptured by BMI, malnutrition status, routine biomarkers, adiposity indices, or spirometry recovery. Malnutrition was reduced by personalized nutritional support but persisted in many patients, supporting more systematic muscle-health and nutritional assessment to allow reduced post-transplant morbidity.

## 1. Introduction

Sarcopenia, defined as a decline in skeletal muscle mass, strength, and potentially physical performance, is increasingly recognized as a clinically relevant syndrome with major implications in the context of aging and chronic diseases [1]. Its prevalence among community-dwelling adults is estimated at approximately 10–20% and rises significantly with advancing age and multimorbidity [2]. Beyond the community setting, sarcopenia is also highly prevalent in chronic disease contexts and has been consistently linked to frailty, impaired functional capacity, and adverse clinical outcomes [1]. In chronic obstructive pulmonary disease (COPD), sarcopenia affects up to one-third of patients, reflecting the combined effects of systemic inflammation, corticosteroid exposure, malnutrition, and physical deconditioning [3]. Sarcopenia has also been associated with impaired quality of life and adverse outcomes in patients with peripheral artery disease [4].

In the field of solid organ transplantation, sarcopenia has been described in several recipient populations—notably heart, liver, and kidney transplant recipients [5,6,7]—where it has been associated with frailty, postoperative complications, and reduced survival. Among lung transplant candidates, the prevalence of sarcopenia is estimated at between 6% and 13% and has been linked to frailty, pre-transplant functional impairment, and an increased risk of removal from the waiting list or death [8]. Existing studies on lung transplantation reveal considerable heterogeneity in the assessment of skeletal muscle status. Imaging-based approaches in lung transplant recipient populations have often relied on skeletal muscle morphometric measurements derived from computed tomography (CT) [9,10]. Functional assessments of muscle strength and performance have also been investigated [11,12], and biomarker-based indices of muscle wasting have been proposed [13,14]. Reduced skeletal muscle mass, as measured by CT, has also been associated with adverse outcomes following lung transplantation [15,16]. However, relatively few studies have applied standardized diagnostic frameworks that integrate both muscle mass and function, such as the criteria of the European Working Group on Sarcopenia in Older People 2 (EWGSOP2) [17]. Only a limited number of studies have assessed sarcopenia using standardized diagnostic frameworks within lung transplant recipient populations [8].

Malnutrition is common in chronic diseases, particularly advanced lung conditions, and is associated with impaired quality of life and poorer clinical outcomes. Given the potential overlap with sarcopenia—driven by inflammation, reduced intake, inactivity, and disease-related catabolism—the relationship between malnutrition and sarcopenia in lung transplant patients warrants further investigation [18,19]. Although nutritional status may improve after transplantation, weight gain does not necessarily reflect a restoration of skeletal muscle mass or function [20]. Consequently, changes in body composition post-transplantation may go clinically unnoticed when assessment relies primarily on weight or body mass index. Furthermore, prolonged corticosteroid exposure, physical inactivity, recurrent illness, and metabolic complications can further hinder skeletal muscle recovery following the procedure [20]. As a result, conventional nutritional and respiratory monitoring measures may not adequately capture the status of muscle health in this population. Notably, data regarding lung transplantation remain relatively limited, even though recipients are exposed to a significant burden of risk factors, such as pre-transplant malnutrition, prolonged immobilization, systemic inflammation associated with advanced lung disease, and chronic corticosteroid exposure [20,21]. To further investigate malnutrition, the framework established by the Global Leadership Initiative on Malnutrition (GLIM) emphasizes both phenotypic and etiologic criteria to characterize impaired nutritional status [18,22].

Several studies have examined the impact of muscle mass loss on patient outcomes following lung transplantation, yielding conflicting results. Reduced muscle mass has been associated with increased perioperative morbidity and mortality in some cohorts [15,16], whereas other studies have not confirmed these associations [23,24,25]. Functional impairments, notably reduced muscle strength and exercise capacity, have also been linked to delayed recovery after transplantation [12,26]. Furthermore, a “sarcopenic overweight” phenotype has been described in lung transplant recipients, highlighting the limitations of body mass index as a marker of muscle health [27,28]. Sarcopenia and post-transplant malnutrition have also been described in the context of critical illness, further underscoring the burden of malnutrition and muscle mass loss in this population [29,30]. Sarcopenia and malnutrition frequently coexist in lung transplant candidates and recipients, yet they represent distinct clinical entities. While sarcopenia specifically refers to the loss of skeletal muscle mass and function, malnutrition encompasses broader nutritional and metabolic disturbances. Given that sarcopenia and malnutrition share common determinants, assessing them simultaneously may be clinically relevant.

This study therefore aimed to determine the prevalence of sarcopenia and malnutrition in a contemporary cohort of lung transplant recipients using recently validated tools: the EWGSOP2 and GLIM criteria. We also examined clinical, biological, and nutritional correlates and evaluated their potential association with post-transplant lung function.

## 2. Population and Methods

### 2.1. Patient Population and Study Design

We conducted a cross-sectional study involving ninety-nine lung transplant recipients followed at the Strasbourg University Hospitals transplant center. All patients presenting for a post-transplant pulmonary follow-up consultation in the outpatient clinic between March and September 2024 were included.

Sarcopenia was assessed at a single time point, using the criteria of the European Working Group on Sarcopenia in Older People 2 (EWGSOP2) [17]. Body composition was assessed using whole-body dual-energy X-ray absorptiometry (DXA) (Discovery DXA system; Hologic Inc., Bedford, MA, USA) in accordance with the manufacturer’s instructions. All scans were performed by the same trained technician.

Malnutrition was assessed at two time points. Pre-transplant nutritional status was determined from medical records documenting a clinical diagnosis of malnutrition or a formal nutritional intervention prior to transplantation. Post-transplant malnutrition was assessed at the time of the study by applying the GLIM criteria.

The study was approved by the Strasbourg institutional ethics committee (reference number: CE-2026-65), and written informed consent was obtained from all participants.

### 2.2. Parameters Determined

#### 2.2.1. Clinical and Biological Characteristics of the Population

Clinical and demographic variables were extracted from the electronic medical record and included age, sex, underlying lung disease, smoking history, time since transplantation, and immunosuppressive therapy, as the prescribed daily dose at the time of sarcopenia assessment.

Laboratory biomarkers were measured in the central laboratory of the University Hospital of Strasbourg using automated chemistry and immunoassay analyzers (Cobas Pro system, Roche Diagnostics, Basel, Switzerland). Measured biomarkers included hemoglobin, serum albumin, C-reactive protein, estimated glomerular filtration rate.

#### 2.2.2. Sarcopenia Assessment

Muscle strength was measured using handgrip dynamometry (Jamar dynamometer, Patterson Medical, Warrenville, IL, USA). Low muscle strength was defined according to EWGSOP2 thresholds (<27 kg for men and <16 kg for women) [17]. A five-times sit-to-stand test (5STS) was also performed when feasible, and a duration greater than 15 s was considered indicative of reduced muscle strength [17].

Muscle quantity was assessed using dual-energy X-ray absorptiometry–derived appendicular lean mass indexed to height squared (ALM/height^2^). Low appendicular lean mass was defined as <7.0 kg/m^2^ in men and <5.5 kg/m^2^ in women.

Participants were classified as sarcopenic when both low muscle strength and low muscle mass were present. Severe sarcopenia was defined when these criteria were accompanied by impaired physical performance, defined as a Short Physical Performance Battery score ≤ 8 [17].

According to the EWGSOP2 diagnostic algorithm (Table 1), assessment began with SARC-F screening. Participants with a SARC-F score ≥ 4 or with clinically suspected muscle weakness proceeded to confirmatory testing. Participants meeting the criterion for low muscle strength were then assessed for low muscle mass using DXA-derived appendicular lean mass. The combination of low muscle strength and low muscle mass established the diagnosis of sarcopenia. Physical performance was subsequently evaluated using the Short Physical Performance Battery; a score of ≤8 was used to define severe sarcopenia.

#### 2.2.3. Nutritional Status

Since using BMI alone likely results in miscalculation of a patient’s true nutrition status [19], the nutritional status was evaluated according to the Global Leadership Initiative on Malnutrition framework (Table 2), incorporating phenotypic criteria such as low body mass index, unintentional weight loss, and reduced muscle mass, and etiologic criteria including reduced food intake and disease burden [18,22]. Recorded nutritional variables included body mass index, documented history of pre-transplant malnutrition requiring nutritional intervention, and post-transplant nutritional status.

Operationally, malnutrition was diagnosed when at least one phenotypic criterion and at least one etiologic criterion were simultaneously met. Phenotypic criteria applied in this study included low body mass index (<18.5 kg/m^2^ regardless of age, or <20 kg/m^2^ in recipients aged ≥70 years), documented unintentional weight loss (>5% within six months, or >10% over a longer period), and DXA-confirmed low appendicular lean mass falling below EWGSOP2 sex-specific thresholds. The etiologic criterion of disease burden was considered met for all recipients given the chronic and systemic nature of end-stage lung disease and long-term immunosuppressive therapy.

Prior to transplantation, all patients underwent at least one nutritional assessment during their hospitalization in the pulmonology department, following the pre-transplant evaluation. All cases were discussed by a multidisciplinary team. This nutritional care is offered to all lung transplant candidates, regardless of age, the severity of respiratory failure, or the underlying respiratory disease.

Fat mass index was calculated as fat mass divided by height squared (kg/m^2^). The adiposity-to-lean mass ratio was computed as fat mass index divided by appendicular lean mass.

#### 2.2.4. Pulmonary Function

Pulmonary function testing was performed using a MasterScreen PFT Pro spirometry system (Jaeger, CareFusion, Hoechberg, Germany) in accordance with American Thoracic Society and European Respiratory Society spirometry standards. Pre-transplant pulmonary function values were obtained from the last spirometry performed before transplantation. The post-transplant baseline forced expiratory volume in one second was defined as the mean of the two highest values recorded after transplantation and before the study assessment visit. Time since transplantation was defined as the interval between the transplantation date and the study assessment visit. On the day of study assessment, corresponding to the DXA and sarcopenia evaluation, patients underwent complete pulmonary function testing, including forced expiratory volume in one second and forced vital capacity.

#### 2.2.5. Statistical Analysis

Statistical analysis was performed using Prism 8.4.3 (Graph Pad Software Inc., San Diego, CA, USA). For quantitative variables, if values were not distributed normally (using the Shapiro–Wilk test), we performed a non-parametric test (Mann–Whitney test). If values were distributed normally, we performed an unpaired *t* test. For non-quantitative variables Fisher’s exact test was performed. Finally, for FEV_1_ evolution, the values were paired and did not follow a normal distribution, we performed a Friedman test. Values are expressed as mean ± standard deviation (SD), or n (%), as appropriate. A *p* value < 0.05 was considered significant.

## 3. Results

### 3.1. Clinical, Biological and Body Composition Characteristics of the Population

Clinical, laboratory, and body composition characteristics according to sarcopenia status are presented in Table 3. The study included 99 lung transplant recipients. The primary diagnosis leading to transplantation were chronic obstructive pulmonary disease (COPD; *n* = 58, 58.6%), cystic fibrosis (CF; *n* = 24, 24.2%), interstitial lung disease (ILD; *n* = 16, 16.2%), and pulmonary arterial hypertension (PAH; *n* = 1, 1.0%).

The mean age of the Ltx was around 60 years, 47% patients were women and their BMI was around 24 kg/m^2^. 70.7% of the patients were smokers and 43% presented with diabetes. The mean glomerular filtration rate was impaired (63.3 mL/min/1.73 m^2^).

Besides corticosteroids, the immunosuppressive regimen associated with lung transplantation included tacrolimus in 92 patients, mycophenolate mofetil in 84 patients and, 11 transplant recipients received azathioprine.

### 3.2. Sarcopenia Was Associated with Age, COPD and ILD, Smoking and Corticoids Exposures

Almost all clinical and biological parameters did not differ significantly between sarcopenic and non-sarcopenic Ltx (Table 3).

**Figure 1:**
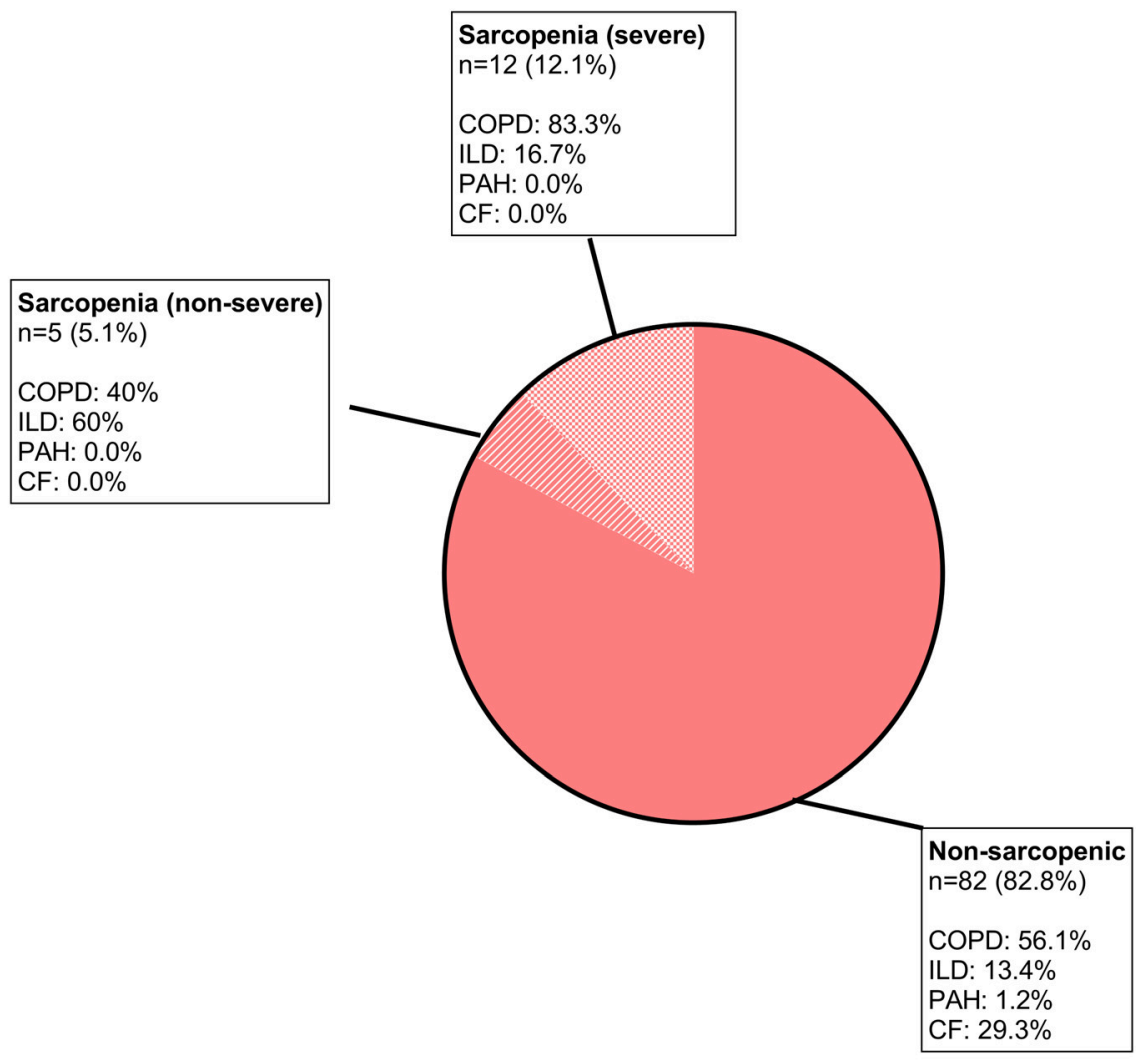
Sarcopenic lung transplant recipients presented mainly with previous COPD and ILD.

The prevalence of sarcopenia was linked to the underlying diseases leading to lung transplantation. Thus, sarcopenia was identified in 17 recipients (17.2%), including 12 (12.1%) classified as having severe sarcopenia and it was observed exclusively among recipients transplanted for COPD and ILD; no cases were identified among recipients transplanted for CF. Among the 12 recipients with severe sarcopenia, 10 were transplanted for COPD (83.3%) and 2 for ILD (16.7%). All 5 non-severe sarcopenia cases were observed in recipients transplanted for COPD (n = 2, 40%) and ILD (n = 3, 60%) (Figure 1).

Pie chart showing the proportion of recipients according to sarcopenia status (n = 99). Sarcopenia was defined according to the EWGSOP2 criteria. Non-severe sarcopenia was observed in recipients transplanted for interstitial lung disease; severe sarcopenia was observed predominantly in recipients transplanted for COPD (83.3%) and ILD (16.7%) No cases of sarcopenia were identified among recipients transplanted for cystic fibrosis.

COPD, chronic obstructive pulmonary disease; CF, cystic fibrosis; ILD, interstitial lung disease; PAH, pulmonary arterial hypertension; EWGSOP2, European Working Group on Sarcopenia in Older People 2.

Sarcopenic recipients tended to be older than non-sarcopenic recipients (66.1 ± 6.7 vs. 57.7 ± 12.6 years; *p* = 0.05).

**Figure 2:**
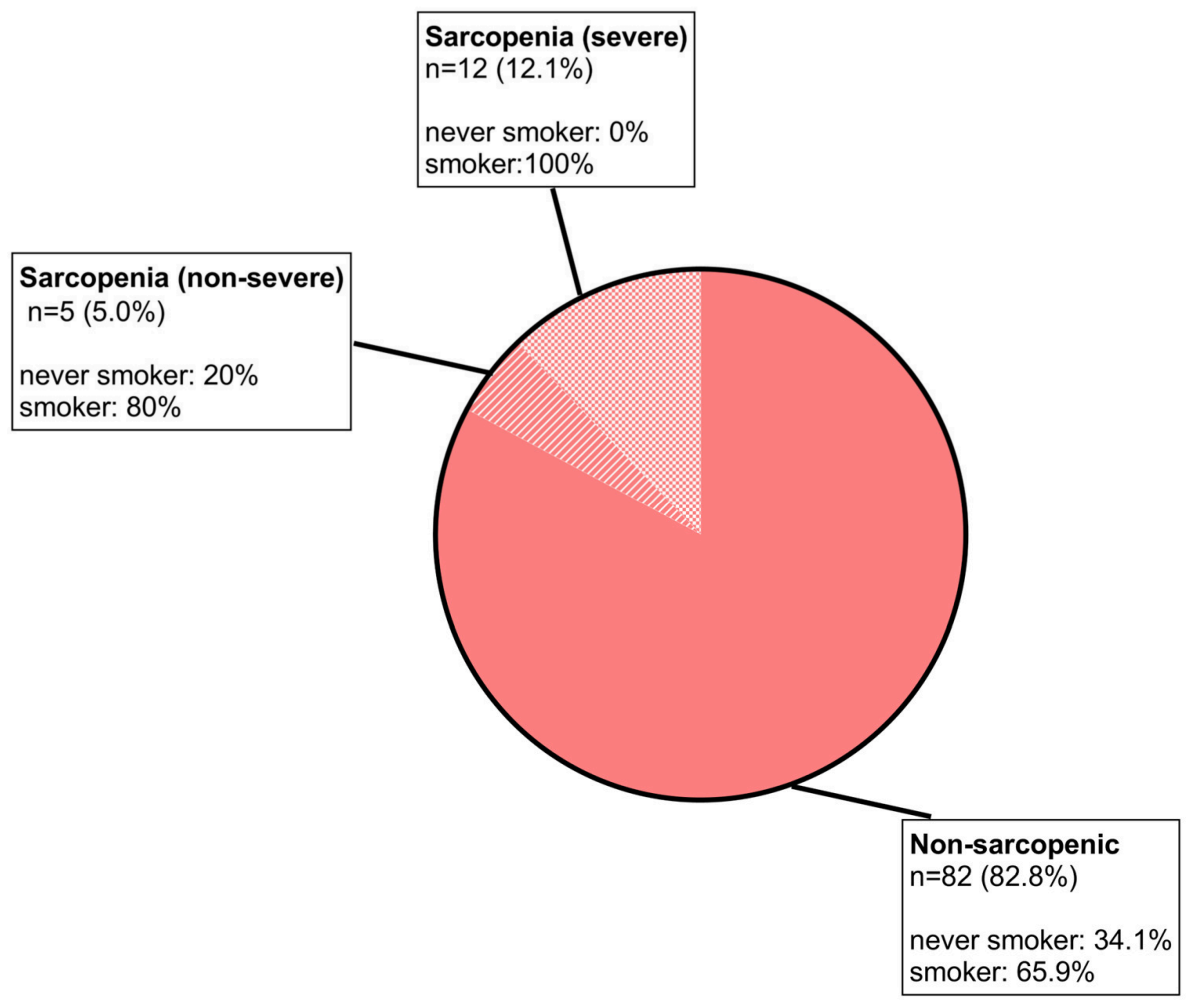
Sarcopenic lung transplant recipients are mainly smokers.

Smoking exposure significantly differed according to sarcopenia status: sarcopenic recipients more frequently reported a prior smoking history (94.1% vs. 62.8%; *p* = 0.02) and had greater cumulative exposure (38.2 ± 25.6 vs. 22.2 ± 20.2 pack-years; *p* = 0.02). Severe sarcopenia was mainly observed in smoker patients (Figure 2).

Pie chart showing the proportion of recipients according to sarcopenia status (n = 99), with the distribution of smoking history within each group. Former smoking was more frequent among sarcopenic recipients than among non-sarcopenic recipients. Sarcopenia was defined according to the EWGSOP2 criteria. EWGSOP2, European Working Group on Sarcopenia in Older People 2.

**Figure 3:**
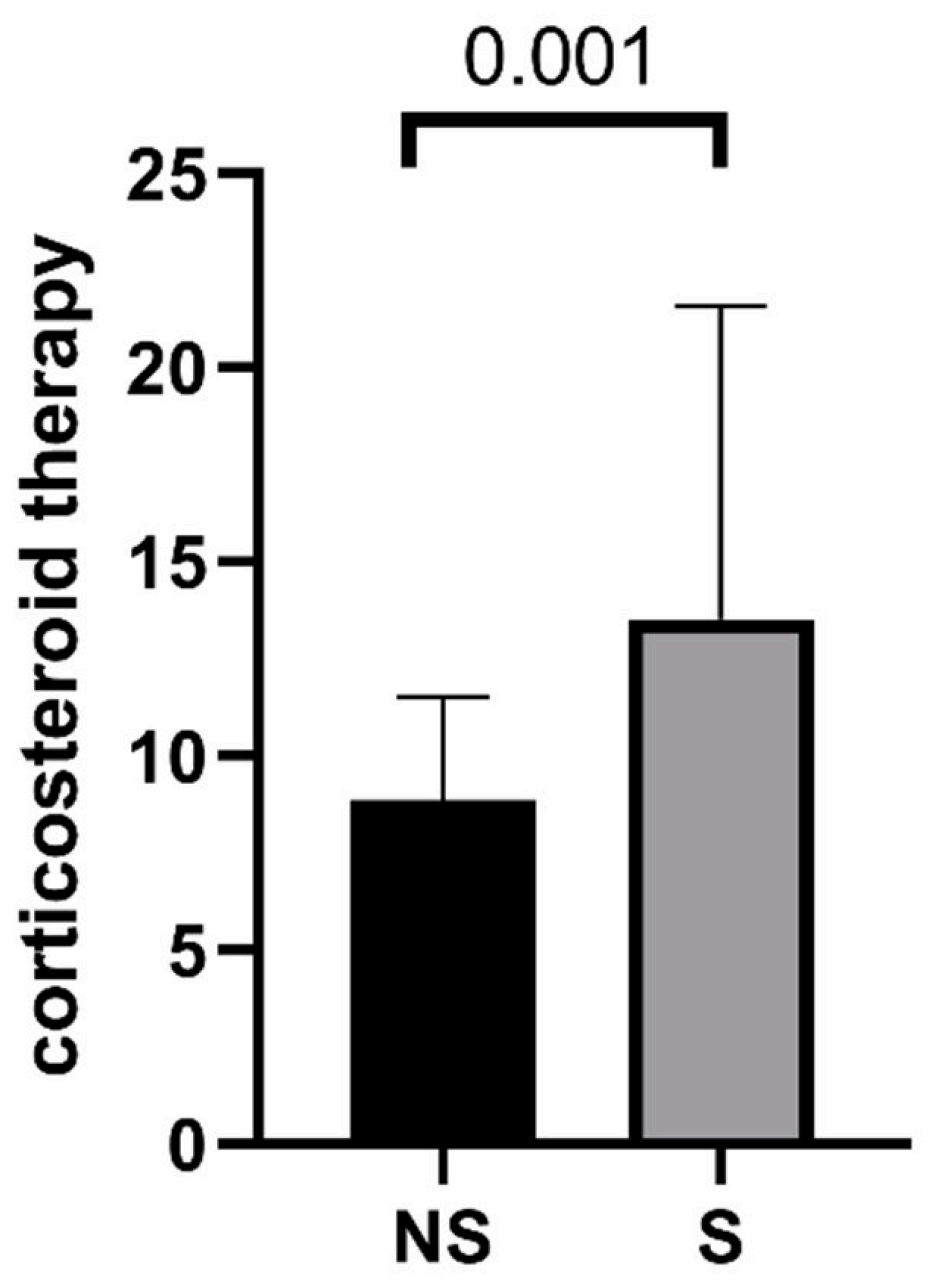
Daily prednisone dose is increased in sarcopenic Ltx.

Finally, at the time of assessment, prescribed daily prednisone dose was significantly higher in sarcopenic recipients than in non-sarcopenic recipients (13.5 ± 8.1 vs. 8.9 ± 2.7 mg/day, respectively; *p* = 0.001, Figure 3).

Bars show mean ± SD prescribed daily prednisone dose. NS, non-sarcopenic recipients; S, sarcopenic recipients. NS group, n = 82; S group, n = 17. Sarcopenic recipients had significantly higher prednisone dose than non-sarcopenic recipients.

The dosages of other immunosuppressive drugs did not differ between lung transplant recipients without sarcopenia and those with sarcopenia (Table 4).

### 3.3. Prevalence of Malnutrition and Associated Factors

**Figure 4:**
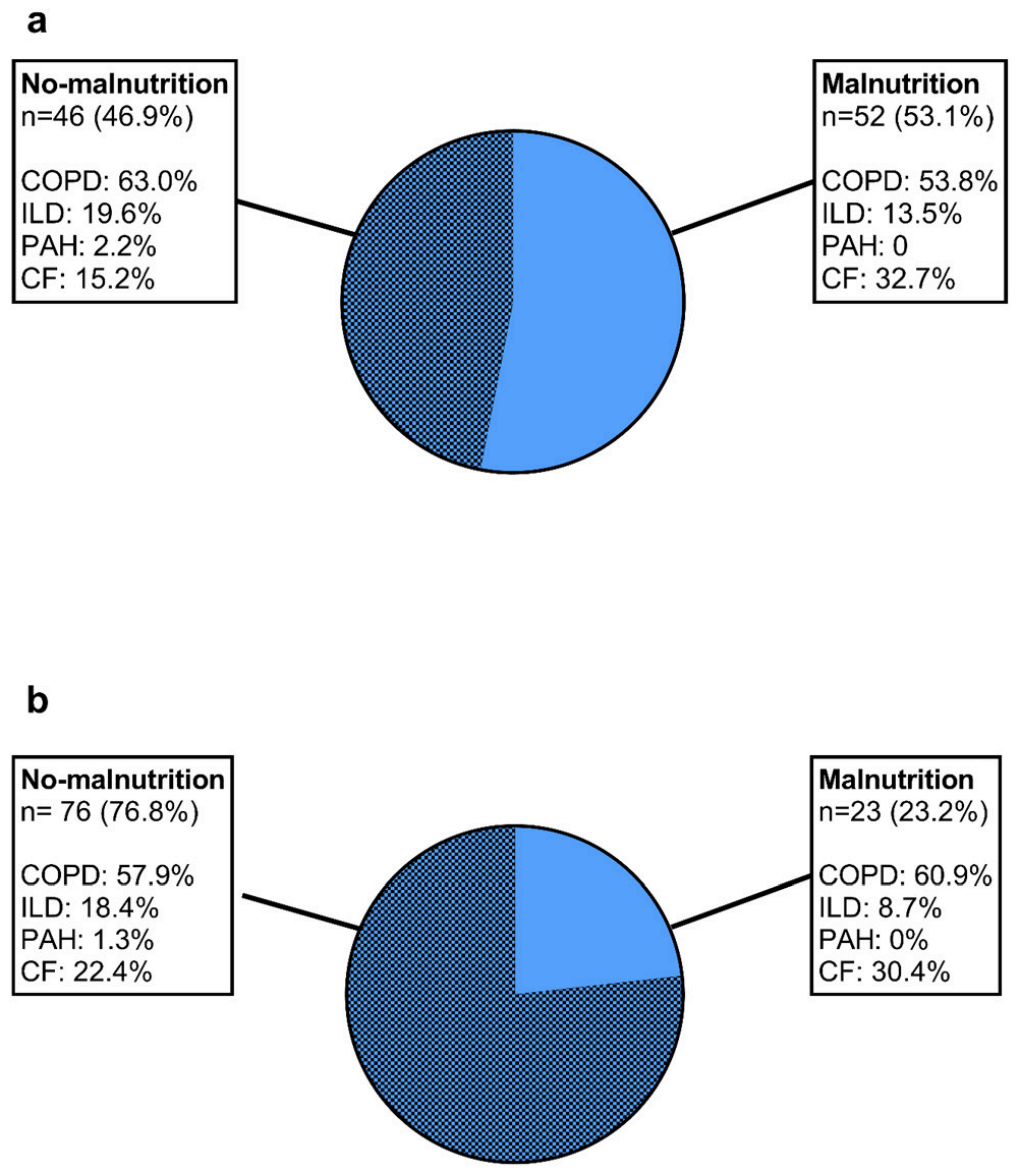
Prevalence of malnutrition before and after lung transplantation, according to the underlying diseases leading to lung transplantation. (**a**) Before lung transplantation; (**b**) After lung transplantation.

Malnutrition was present both before and after transplantation. Pre-transplantation nutritional data were available for 98 recipients. Post-transplantation nutritional status was assessed in all 99 recipients. Pre-transplantation malnutrition was observed in 52 patients (53.1%), whereas 23 patients (23.2%) exhibited malnutrition during post-transplantation follow-up, indicating that malnutrition persisted in nearly a quarter of recipients after the procedure (Figure 4a). Recipients suffering from malnutrition before transplantation were more likely to remain malnourished afterwards than those who were not initially malnourished (36.5% vs. 8.7%).

Among malnourished recipients, COPD accounted for the majority of cases both before (53.8%) and after transplantation (60.9%)—reflecting the prevalence of this condition within the cohort—followed by cystic fibrosis (32.7% and 30.4%) and diffuse interstitial lung diseases (13.5% and 8.7%) (Figure 4b).

Pie chart showing in 4a, the prevalence of malnutrition before lung transplantation, according to their underlying diseases. Figure 4b present the percentage of Ltx without and with malnutrition after lung transplantation. Malnutrition was defined according to the GLIM criteria. COPD, chronic obstructive pulmonary disease; CF, cystic fibrosis; ILD, interstitial lung disease; PAH, pulmonary arterial hypertension; GLIM, Global Leadership Initiative on Malnutrition.

To further the analysis, we characterized patients based on the presence or absence of malnutrition at the time of DXA assessment. Table 5 shows that many of the typical clinical and biological characteristics of lung transplant recipients are not associated with malnutrition. However, DXA analysis revealed a significant reduction in fat mass parameters in malnourished patients compared to those who were not malnourished.

Interestingly, immunosuppressant dosages did not differ significantly in lung transplant patients in the absence or presence of malnutrition (Table 6).

### 3.4. Potential Link Between Nutritional Status and Sarcopenic Status

**Figure 5:**
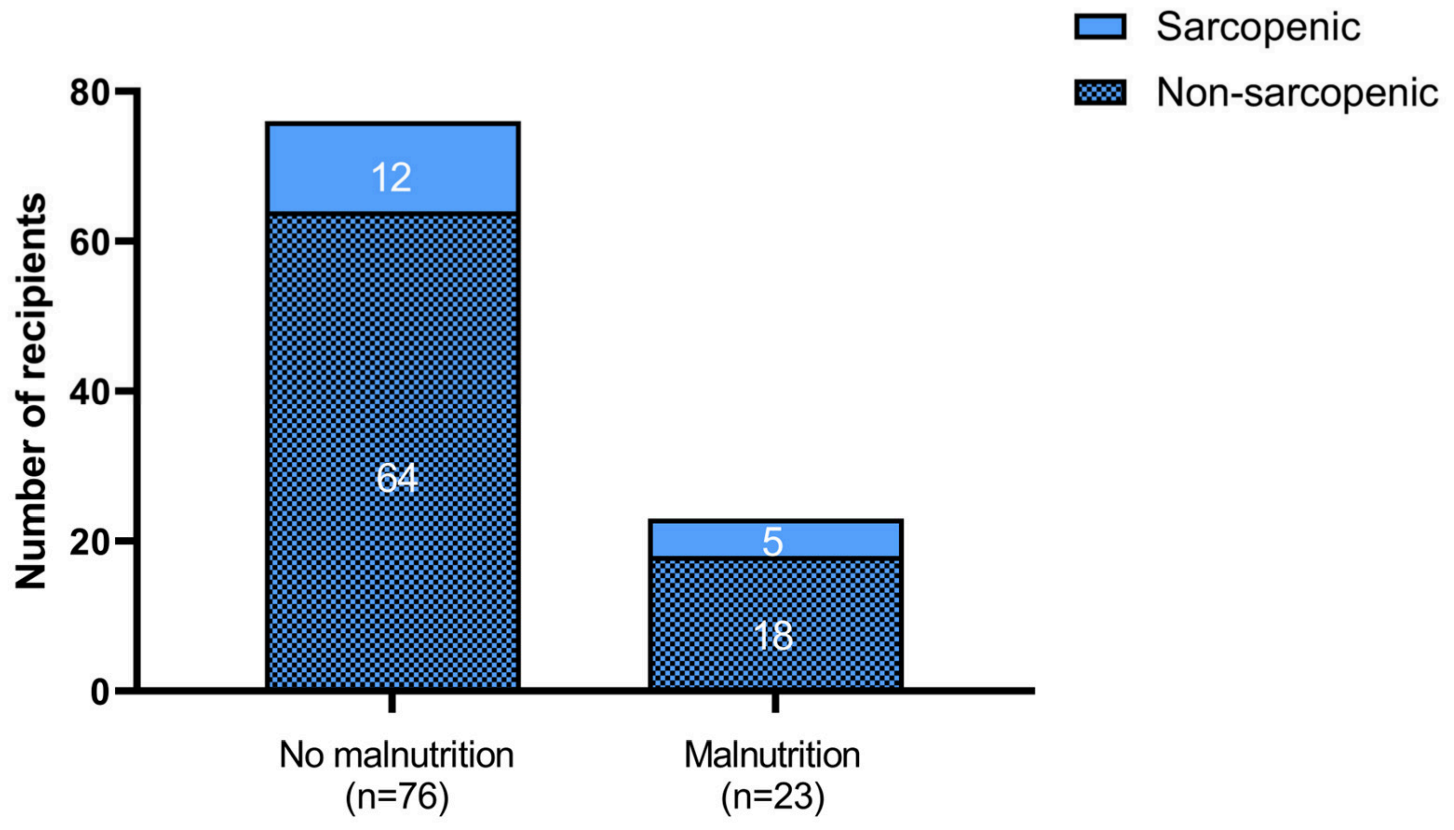
Sarcopenia prevalence did not differ significantly in lung transplant recipients presenting without or with malnutrition.

Since sarcopenia might be associated with malnutrition, we determined the number of sarcopenic patients in both Ltx presenting without and with malnutrition. Although albuminemia tended to be decreased in sarcopenic Ltx (*p* = 0.07), the number of sarcopenic Ltx was not significantly increased in the malnourished patients (*p* = 0.53) (Figure 5).

Among recipients without post-transplant malnutrition (n = 76), 64 were non-sarcopenic and 12 were sarcopenic. Among recipients with post-transplant malnutrition (n = 23), 18 were non-sarcopenic and 5 were sarcopenic. Sarcopenia was defined according to EWGSOP2 criteria. Malnutrition was defined according to GLIM criteria. Abbreviations: EWGSOP2, European Working Group on Sarcopenia in Older People 2; GLIM, Global Leadership Initiative on Malnutrition; LTx, lung transplantation.

### 3.5. Pulmonary Function in Relation to Sarcopenia

**Figure 6:**
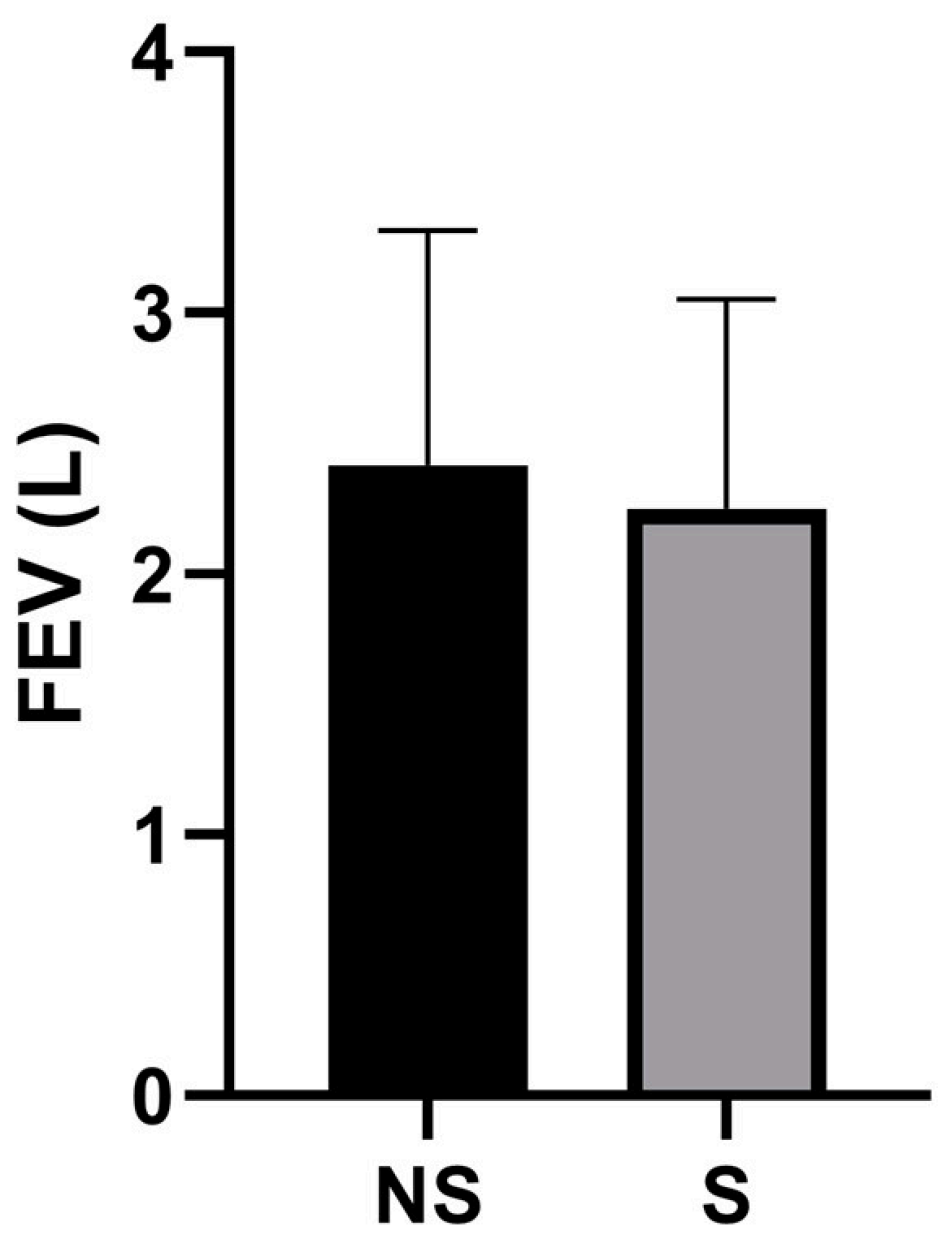
Mean FEV_1_ was similar in non- and in sarcopenic Ltx.

To investigate a potential impaired pulmonary function associated with sarcopenia, we analyzed FEV_1_ measurements performed on 89 patients prior to transplantation and two times after Ltx. The pulmonary function improved significantly after transplantation across the cohort compared to the pre-transplantation value (0.78 ± 0.05 vs. 2.82 ± 0.09 L, preLTX vs. baseline respectively, *p* < 0.0001), Figure 6. At the time of the DXA scan FEV_1_ decreased moderately but significantly across the entire cohort—14.3% (2.42 ± 0.09 L, *p* < 0.0001).

After transplantation, using DXA, we compared sarcopenic and non-sarcopenic groups. The results show no significant difference in FEV_1_ between the two groups at the time of the DXA scan (2.42 ± 0.1 L vs. 2.25 ± 0.19 L, for non-sarcopenic and sarcopenic group respectively).

Mean forced expiratory volume in one second (FEV_1_) The measurement was taken at the time of dual-energy X-ray absorptiometry (DXA) assessment so post-transplant. Sarcopenia was defined according to the European Working Group on Sarcopenia in Older People 2 (EWGSOP2) criteria. Values are expressed as mean ± SD. n = 17 in sarcopenic group and n = 82 in non-sarcopenic group. FEV_1_, forced expiratory volume in one second; LTx, lung transplantation; DXA, dual-energy X-ray absorptiometry; EWGSOP2, European Working Group on Sarcopenia in Older People 2.

## 4. Discussion

This study evaluated the prevalence of sarcopenia and malnutrition in lung transplant recipients using recently validated tools: the EWGSOP2 and GLIM criteria. Sarcopenia was observed in 17 (17.2%) of the 99 recipients, with 12 presenting a severe form. Patients with sarcopenia primarily suffered from COPD or interstitial lung disease (ILD); they were older and had a higher history of smoking and higher prednisone dosages than non-sarcopenic patients. BMI, adiposity indices, standard biomarkers, and spirometric parameters were comparable between patients with and without sarcopenia. Malnutrition was common prior to transplantation (53.1%) and persisted in nearly a quarter (23.2%) of patients, primarily those with COPD or cystic fibrosis. This marked improvement was likely linked to nutritional support, ranging from dietary advice to enteral or parenteral nutrition. Despite a trend toward lower serum albumin levels, sarcopenia was not significantly associated with malnutrition as defined by GLIM criteria.

### 4.1. Sarcopenia in Lung Transplant Recipients

Sarcopenia is increasingly recognized as a clinically significant syndrome in aging populations and in the context of chronic diseases [1,2,3,4]. In the field of solid organ transplantation, impaired muscle mass and performance have been linked to frailty, reduced functional capacity, and adverse post-transplant outcomes [5,6,7,31]. Most previous studies on lung transplantation have relied on CT-based morphometric measurements to estimate skeletal muscle mass or body composition [9,10]. Although these approaches provide information on muscle quantity, they do not fully capture the multidimensional nature of sarcopenia, which encompasses muscle quantity, strength, and physical performance. The present study applied the EWGSOP2 definition, allowing for a more comprehensive characterization of skeletal muscle status in lung transplant recipients [17]. Indeed, the clinical value of combining DXA-based muscle mass measurement with grip strength has also been reported in chronic inflammatory myopathies, where this phenotype was associated with disability, disease-related damage, and myokine imbalance [32].

In our study, sarcopenia was found to be relatively common among lung transplant recipients evaluated during follow-up; however, it showed no association with the numerous clinical, biological, and respiratory parameters typically used for monitoring. These standard parameters may therefore fail to adequately identify recipients with impaired muscle mass or function. Nevertheless, the underlying condition necessitating transplantation, age, higher cumulative tobacco exposure, and higher prednisone doses were associated with sarcopenia.

Differences in sarcopenia prevalence were observed based on the indication for transplantation. Sarcopenia was detected in recipients transplanted for COPD or diffuse interstitial lung disease (ILD), whereas no cases were identified among those transplanted for cystic fibrosis or pulmonary hypertension. This pattern likely reflects the younger age and distinct clinical trajectory of cystic fibrosis recipients, who generally undergo transplantation at an earlier stage of systemic decline compared to patients with obstructive or fibrotic lung diseases. Furthermore, it is well established that COPD and ILD are associated with muscle alterations that may predispose patients to sarcopenia following lung transplantation [33,34,35,36]. No conclusions can be drawn regarding pulmonary hypertension, given that only one patient with this condition was included in the study, even though muscle alterations have also been observed in this context [37].

Interestingly, sarcopenic recipients were receiving higher daily doses of prednisone than non-sarcopenic recipients. This finding is not surprising, as increased exposure to corticosteroids can contribute to muscle alterations by inhibiting muscle protein synthesis and/or exerting catabolic effects on skeletal muscle [38,39,40]. In contrast, no significant relationship was observed between sarcopenia in lung transplant recipients and their other immunosuppressive treatments.

The observed link between tobacco exposure and sarcopenia—whether based on smoking history or cumulative smoking burden (expressed in pack-years)—aligns with the established role of smoking as a risk factor for skeletal muscle dysfunction, mediated by systemic inflammation, oxidative stress, and alterations in protein degradation and vascular signaling pathways [41,42,43,44].

Although this finding is consistent with a previous study [45], sarcopenia was not associated with significantly impaired lung function in lung transplant recipients compared to those without sarcopenia. This result warrants confirmation in a larger cohort undergoing longitudinal follow-up; it also suggests that a more specific assessment of respiratory muscles might be necessary. In this context, it would have been valuable to assess post-transplant exercise capacity—for instance, using the 6 min walk test—to investigate a potential association between sarcopenia and reduced walking distance, a factor that could also be linked to diminished quality of life and/or increased mortality [46,47].

### 4.2. Malnutrition Before and After Lung Transplantation

Consistent with previous data based on GLIM criteria [48], malnutrition was common in this cohort, both before and after transplantation. Pre-transplant malnutrition was documented in slightly more than half of the recipients (53.1%); this aligns with findings by Calanas-Continente et al., who reported malnutrition in 47% of lung transplant candidates [18], as well as with the high nutritional burden associated with advanced lung disease. Hypermetabolism and caloric deficit, combined with loss of appetite and impaired gastrointestinal function, contribute to malnutrition [18,49,50,51,52]. Notably, a reduction in lean body mass is associated with a poorer clinical status and a worse prognosis in these patients [53].

To address this malnutrition, we tailored nutritional management for the patients. Patients with a BMI below 18.5 kg/m^2^ received oral nutritional supplements, with or without enteral nutrition. For those with a BMI between 18.5 and 30 kg/m^2^, only dietary counseling was provided. Patients with a BMI > 30 kg/m^2^ received advice on adopting a balanced diet—combined, in some cases, with an exercise rehabilitation program—to achieve gradual, controlled weight loss [54], for review [55].

It is noteworthy that nearly a quarter of recipients remained malnourished at the follow-up assessment. Recipients who were malnourished prior to transplantation were significantly more likely to remain malnourished after the procedure than those who were well-nourished beforehand (36.5% vs. 8.7%), highlighting the prognostic importance of pre-transplant nutritional status. The distribution of malnutrition across diagnostic groups remained relatively stable before and after transplantation, with COPD recipients consistently accounting for the largest proportion. Recipients with cystic fibrosis represented 32.7% of malnourished patients before transplantation and 30.4% at follow-up; this may reflect the persistence of the disease’s systemic and gastrointestinal manifestations after transplantation [56]. In contrast, malnutrition was less common at follow-up among recipients with interstitial lung disease (8.7%)—a group in which nutritional improvement following the elimination of the underlying disease’s inflammatory and restrictive burden might be more pronounced.

The marked postoperative improvement likely resulted from the precision of the patient’s nutritional assessment and support—an approach justified by the fact that malnutrition at the time of lung transplantation is associated with a poorer prognosis. Thus, improving nutritional status remains a major clinical goal [57]. Consequently, following transplantation, an initial nutritional assessment was performed in the intensive care unit within the first few days after the procedure. Parenteral nutrition was frequently used, although enteral nutrition could also be employed. A second nutritional assessment was conducted after transferring to the pulmonary intensive care unit, generally around the seventh postoperative day. At this stage, the primary goal was usually to discontinue parenteral nutrition, while ensuring a transition to enteral nutrition if necessary. Subsequently, nutritional reassessments were performed every two days throughout the post-transplant hospital stay (including body weight measurement and assessment of 24 h dietary intake via consumption records) to adjust caloric intake and artificial nutritional support as needed. After hospital discharge, a significant proportion of patients participated in a pulmonary rehabilitation program, with nutritional monitoring provided by the rehabilitation facility. A systematic nutritional reassessment was also performed during the first outpatient follow-up visit, generally 10 to 15 days after discharge from the pulmonary unit. Further nutritional interventions were implemented at the request of the attending physicians and/or the patients.

### 4.3. Potential Relationship Between Sarcopenia and Malnutrition in Lung Transplant Recipients

In this cross-sectional study, neither sarcopenia status nor pre-transplant malnutrition predicted the other parameter. Pre-transplant malnutrition was observed in 10 of the 17 sarcopenic recipients (58.8%) and in 42 of the 81 non-sarcopenic recipients (51.9%). Pre-transplant malnutrition was not significantly associated with sarcopenia status. Post-transplant malnutrition was present in 5 of the 17 sarcopenic recipients (29.4%) and in 18 of the 82 non-sarcopenic recipients (22.0%); here again, no significant association was observed between post-transplant malnutrition and sarcopenia.

These results suggest that sarcopenia and malnutrition may represent partially independent dimensions of impaired nutritional and muscular status in this population, each warranting specific assessment rather than the use of one as a surrogate marker for the other. Conventional nutritional markers, such as BMI and serum albumin, were comparable between sarcopenic and non-sarcopenic recipients, confirming the limitations of these indicators in reflecting skeletal muscle status. Interestingly, no difference was found regarding the fat mass-to-lean mass ratio between sarcopenic and non-sarcopenic patients. This finding is likely attributable to several factors, notably the screening tools used (in the context of malnutrition screening based on GLIM criteria, an etiological criterion is systematically met for all recipients, whereas EWGSOP2 criteria may more accurately reflect the extent of functional muscle activity and muscle status).

Nevertheless, hypoalbuminemia is a classic marker of malnutrition. In our study populations, we observed a trend toward lower serum albumin levels in sarcopenic patients (*p* = 0.07) compared with non-sarcopenic lung transplant recipients. Thus, future studies could better elucidate the link between sarcopenia and malnutrition in lung transplant recipients, as has previously been observed in kidney transplant recipients [58].

Overall, these results suggest that incorporating a standardized assessment of muscle health and malnutrition into the follow-up of lung transplant recipients could help identify recipients with functional vulnerability that is not detectable via conventional nutritional or respiratory markers. Simple functional assessments—such as grip strength measurement or the chair-stand test—combined with periodic body composition assessment, could help identify recipients at risk of functional decline who might otherwise go undetected by routine anthropometric or biological monitoring.

### 4.4. Study Limitations

The cross-sectional and single-center nature of the study, the relatively small number of patients with sarcopenia, and the absence of multivariate analyses warrant caution regarding the interpretation and generalizability of the results. Nevertheless, these findings were obtained using sarcopenia and malnutrition screening tools applied to a large cohort of lung transplant patients in a real-world setting; they thus provide a clear picture of the situation and enable better screening of high-risk patients, facilitating optimal adjustment of pre- and peri-transplant interventions.

## 5. Conclusions and Future Directions

In this cohort, sarcopenia was present in 17% of lung transplant recipients during routine follow-up and was predominantly severe. The fact that it is independent of established nutritional and respiratory markers highlights a gap in current monitoring practices. Systematic assessment of muscle health following lung transplantation would make it possible to identify recipients with residual functional impairment that conventional screening fails to detect and could, ultimately, guide targeted rehabilitation strategies. Indeed, loss of skeletal muscle mass has been independently associated with reduced overall survival following lung transplantation for interstitial lung disease [59]. Furthermore, although it decreases significantly after transplantation, malnutrition persists in a substantial number of patients, warranting improvements in both the diagnosis and prevention of this deleterious condition. Indeed, nutritional support improves the clinical prognosis of patients undergoing lung transplantation [60]. Longer-term longitudinal studies would be valuable to further investigate the potential association between severe sarcopenia and/or malnutrition and impaired lung function or exercise capacity, as well as to determine the therapeutic potential of physical exercise, nutrition, and/or therapies targeting muscle anabolism.

## Figures and Tables

**Table 1:** EWGSOP2 diagnostic components applied in this study.

Component	Tool/Method	Threshold (Low)
Screening	SARC-F questionnaire	Score ≥ 4
Muscle strength	Handgrip dynamometry (Jamar); 5STS as alternative	<27 kg (men); <16 kg (women); 5STS > 15 s
Muscle mass	DXA-derived ALM/height^2^	<7.0 kg/m^2^ (men); <5.5 kg/m^2^ (women)
Physical performance	Short Physical Performance Battery (SPPB)	Score ≤ 8 (defines severe sarcopenia)

ALM, appendicular lean mass; DXA, dual-energy X-ray absorptiometry; SPPB, Short Physical Performance Battery; 5STS, five-times sit-to-stand test, EWGSOP2, European Working Group on Sarcopenia in Older People 2.

**Table 2:** GLIM diagnostic criteria applied in this study.

Domain	Criterion	Operational Definition in This Study
Phenotypic criteria (≥1 required)	Low BMI	<18.5 kg/m^2^ (any age); <20 kg/m^2^ (age ≥ 70 years)
	Unintentional weight loss	>5% within 6 months, or >10% over any longer period
	Reduced muscle mass	DXA-derived ALM/height^2^ below EWGSOP2 thresholds (<7.0 kg/m^2^ men; <5.5 kg/m^2^ women)
Etiologic criteria (≥1 required)	Reduced food intake/assimilation	Documented pre-transplant nutritional intervention or clinical diagnosis of malnutrition in medical records
	Disease burden/inflammation	Met for all recipients (chronic end-stage lung disease; long-term immunosuppressive therapy)

Diagnosis requires ≥1 phenotypic criterion AND ≥1 etiologic criterion. ALM, appendicular lean mass; BMI, body mass index; DXA, dual-energy X-ray absorptiometry.

**Table 3:** Clinical, laboratory, and body composition characteristics of the population.

Characteristic	Overall (n = 99)	Non-Sarcopenic (n = 82)	Sarcopenic (n = 17)	*p*-Value
**Age, years**	**59.7 ± 12.2**	**57.7 ± 12.6**	**66.1 ± 6.7**	**0.05**
Female sex, n (%)	47 (47.5)	39 (47.6)	8 (47.1)	>0.999
Height, cm	166.4 ± 9.0	166.7 ± 9.1	165.2 ± 8.9	0.54
Weight, kg	65.6 ± 14.8	66.7 ± 15.4	60.0 ± 10.1	0.09
BMI, kg/m^2^	23.6 ± 4.8	24.0 ± 5.0	22.0 ± 3.2	0.25
Time since transplantation, years	6.3 ± 5.3	6.8 ± 5.5	4.3 ± 3.9	0.09
**Smoking status, n** (**%**) **Never/Former**	**29** (**29.3**)**/70** (**70.7**)	**28** (**34.1**)**/54** (**65.9**)	**1** (**5.9**)**/16** (**94.1**)	**0.02**
**Pack-years**	**24.9 ± 21.9**	**22.2 ± 20.2**	**38.2 ± 25.6**	**0.02**
Diabetes, n (%)	43 (43.4)	38 (46.3)	5 (29.4)	0.28
CRP, mg/L	6.2 ± 10.3	6.6 ± 11.0	4.2 ± 4.8	0.87
eGFR, mL/min/1.73 m^2^	63.3 ± 24.3	61.7 ± 24.5	70.9 ± 22.8	0.16
*Albumin, g/L*	*41.8 ± 4.4*	*42.1 ± 4.3*	*40.5 ± 4.7*	*0.07*
Hemoglobin, g/dL	12.4 ± 1.9	12.5 ± 1.8	11.8 ± 2.2	0.20
Fat mass, %	32.9 ± 9.5	32.6 ± 9.7	34.2 ± 9.0	0.52
Fat mass/fat-free mass ratio	0.52 ± 0.22	0.52 ± 0.22	0.54 ± 0.20	0.66
Fat mass index, kg/m^2^	7.9 ± 3.53	7.97 ± 3.67	7.58 ± 2.76	0.90
Adiposity-to-lean ratio (FMI/ALM)	1.37 ± 0.61	1.33 ± 0.60	1.53 ± 0.62	0.21

Values are presented as mean ± SD or n (%), as appropriate. Continuous variables were compared using unpaired *t* tests or Mann–Whitney U tests according to distribution. Categorical variables were compared using Fisher’s exact test. BMI, body mass index; CRP, C-reactive protein; eGFR, estimated glomerular filtration rate; FMI, fat mass index; ALM, appendicular lean mass.

**Table 4:** Immunosuppressive treatment in sarcopenic and non-sarcopenic lung transplant recipients.

Immunosuppressive Therapy	Non Sarcopenic LTx (n = 82)	Sarcopenic LTx (n = 17)	*p*-Value
**Corticosteroid** (mg/day)	**8.87 ± 2.67**	**13.53 ± 8.06**	**0.001**
Tacrolimus (mg/day)	3.92 ± 2.56	3.68 ± 3.29	0.42
Mycophenolate mofetil (mg/day)	1413 ± 832.4	1261 ± 892.9	0.72
Azathioprine (mg/day)	7.93 ± 24.81	20.59 ± 50.18	0.25

**Table 5:** Clinical, laboratory, and body composition characteristics of the population according to the nutritional status.

Patient’s Characteristics	No-Malnutrition (n = 76)	Malnutrition (n = 23)	*p*-Value
Age, years	61.11 ± 11.18	55.96 ± 14.37	0.31
Female sex, n (%)	34 (44.7%)	13 (56.5%)	0.35
Height, cm	166.2 ± 8.8	167.0 ± 10.0	0.73
**Weight, kg**	**69.92 ± 13.78**	**51.17 ± 6.77**	**<0.0001**
**BMI, kg/m^2^**	**25.23 ± 4.21**	**18.32 ± 1.52**	**<0.0001**
Time since transplantation, years	6.06 ± 5.16	7.20 ± 5.89	0.26
Smoking status, n (%) Never/Former	20 (26.3)/56 (73.7)	9 (39.1)/14 (60.9)	0.30
Pack-years	26.37 ± 22.55	20.23 ± 19.36	0.33
Diabetes, n (%)	35 (46.0)	8 (34.8)	0.47
CRP, mg/L	6.26 ± 10.60	5.88 ± 9.21	0.97
eGFR, mL/min/1.73 m^2^	60.92 ± 22.03	71.13 ± 29.94	0.08
*Albumin, g/L*	*41.87* ± 4.52	*41.61* ± *3.92*	*0.39*
Hemoglobin, g/dL	12.48 ± 1.96	12.01 ± 1.67	0.30
**Fat mass, %**	**34.98 ± 8.89**	**25.83 ± 8.18**	**<0.0001**
**Fat mass/fat-free mass ratio**	**0.57 ± 0.21**	**0.36 ± 0.15**	**<0.0001**
**Fat mass index, kg/m^2^**	**8.87 ± 3.35**	**4.69 ± 1.71**	**<0.0001**
**Adiposity-to-lean ratio (FMI/ALM)**	**1.49 ± 0.60**	**0.98 ± 0.44**	**0.0003**

**Table 6:** Immunosuppressive treatment in lung transplant recipients who are well-nourished or malnourished.

Immunosuppressive Therapy	No Malnutrition (n = 76)	Malnutrition (n = 23)	*p*-Value
Corticosteroids (mg/day)	9.41 ± 3.87	10.54 ± 5.93	0.71
Tacrolimus (mg/day)	3.83 ± 2.77	4.02 ± 2.43	0.65
Mycophenolate mofetil (mg/day)	1456 ± 799.7	1159 ± 945.8	0.15
Azathioprine (mg/day)	8.22 ± 27.5	16.30 ± 39.61	0.38

## Data Availability

The data presented in this study are available on request from the corresponding author due to privacy.
